# Inactivation of *Bacillus subtilis* by Curcumin-Mediated Photodynamic Technology through Inducing Oxidative Stress Response

**DOI:** 10.3390/microorganisms10040802

**Published:** 2022-04-12

**Authors:** Li Dong, Jianran Qin, Luyang Tai, Kangyi Mou, Xiaojun Liao, Fang Chen, Xiaosong Hu

**Affiliations:** College of Food Science and Nutritional Engineering, National Engineering Research Center for Fruit and Vegetable Processing, Key Laboratory of Fruits and Vegetables Processing, Ministry of Agriculture, Engineering Research Centre for Fruits and Vegetables Processing, Ministry of Education, China Agricultural University, Beijing 100083, China; li_dong127@163.com (L.D.); qinjianran0504@163.com (J.Q.); 18013885232@126.com (L.T.); mkys1999@163.com (K.M.); liaoxjun@cau.edu.cn (X.L.); chenfangch@sina.com (F.C.)

**Keywords:** curcumin, *B. subtilis*, ROS, DNA damage, oxidative stress

## Abstract

Photodynamic sterilization technology (PDT) is widely used in disease therapy, but its application in the food industry is still at the research stage because of the limitations of food-grade photosensitizers. Curcumin exhibits photosensitivity and is widely used as a food additive for its natural color. This study aimed to determine the effect of curcumin-mediated photodynamic technology (Cur-PDT) on *Bacillus subtilis* and to elucidate the anti-bacterial mechanism involved. First, the effects of curcumin concentration, duration of light irradiation, light intensity, and incubation time on the inactivation of *B. subtilis* were analyzed. It was found that Cur-PDT inactivated 100% planktonic cells with 50 μmol/L curcumin in 15 min (120 W). Then, the cell morphology, oxidation state and the expression of membrane structure- and DNA damage-related genes of *B. subtilis* vegetative cells were investigated under different treatment conditions. The membrane permeability of cells was enhanced and the cell membrane structure was damaged upon treatment with Cur-PDT, which were exacerbated with increases of treatment time and curcumin concentration. Meanwhile, the production of reactive oxygen species increased and the activities of the antioxidant enzymes SOD, GPX, and CAT decreased inside the cells. Furthermore, the Cur-PDT treatment significantly downregulated the mRNA of the membrane protein TasA and upregulated the DNA damage recognition protein UvrA and repair protein RecA of *B. subtilis*. These results suggested that curcumin-mediated PDT could effectively inactivate *B. subtilis* by inducing cell redox state imbalance, damaging DNA, and disrupting membrane structures.

## 1. Introduction

In recent years, with the increasing public demand for nutritious food and a healthy lifestyle, food processing technology has been changing. In this context, increasing attention has been directed at non-thermal sterilization technologies, which can reduce the nutrient loss and flavor damage caused by high temperature [1,2]. However, the existing versions of these technologies, mainly involving ultra-high pressure, plasma treatment, and ultraviolet irradiation, would increase costs in the food industry due to the need for rigid equipment and the production of harmful by-products [1,3,4,5]. Therefore, many researchers have focused on finding effective alternative non-thermal sterilization methods for food preservation.

Photodynamic sterilization technology (PDT) has been widely used in disease therapy. It can effectively kill a variety of pathogenic microorganisms in the presence of oxygen, visible light, and photosensitizer [6,7,8]. Upon illumination with light of a specific wavelength, the photosensitizer can stimulate singlet oxygen and then promote the production of a large amount of reactive oxygen species (ROS), damaging the cell structure and even resulting in cell death through oxidation stress. Owing to the selectivity of its photosensitizer, PDT can effectively inactivate microorganisms without damaging adjacent host cells or tissues [7]. Recent studies have shown that photodynamic sterilization can inactivate *Escherichia coli*, *Listeria monocytogenes*, and other microorganisms, while it is also used in freshly cut fruit, fruit juice, and aquatic products [9,10]. Treatment with hypericin-mediated PDT was reported to reduce the count of *Bacillus cereus* by 1.1, 0.7, and 1.3 log CFU/g on the surfaces of apricot (*Prunus armeniaca*), plum (*Prunus domestica*), and cauliflower (*Brassica oleracea*), respectively [10]. In addition, in an experiment on the application of curcumin-based PDI to freshly cut potato slices, 30 μmol/L curcumin solution reduced the levels of *E. coli* and *Staphylococcus. aureus* by 2.43 log and 3.18 log, respectively, upon exposure to a 420 nm LED [11]. PDT has also been demonstrated to have the advantages of simultaneously killing microorganisms and maintaining the sensory properties (color, flavor, and texture) of processed food [9,12,13,14]. Therefore, PDT is becoming a novel innovative approach for sterilization technology in the food industry.

*Bacillus* is a genus of spore-producing Gram-positive bacteria. In extreme environments, it can produce highly resistant spores that are difficult to kill. Its existence raises great challenges with regard to medical pollution and food corruption [15,16]. For example, *Bacillus. cereus* is often present in starchy and proteinaceous foods, such as bean products and rice flour products, and can produce a toxin that causes vomiting and diarrhea [17]. *Alicyclobacillus* can cause spoilage in in fruit juice [18]. Even, *Bacillus anthracis* has been used as a biological weapon through causing anthrax disease [19]. It is thus important to strictly limit the amount of *Bacillus* in food.

Curcumin, as a natural polyphenolic bioactive compound isolated from *Curcumalonga eugenio* rhizomes, is widely used as a food additive in food processing to maintain color [20]. Because of its photosensitivity, previous studies showed that curcumin can partially inactivate *E. coli* [11], *Vibrio parahaemolyticus* [21], and the spores of *Aspergillus flavus* [22]. Therefore, to obtain a better understanding of the effect of curcumin-mediated PDT on *B. subtilis*, we assessed the sterilizing effects of curcumin under light irradiation and confirmed that ROS-induced oxidant stress actively participated in PDT in *B. subtilis*. Additionally, via the determination of antioxidant enzyme activity, morphological investigation, and evaluation of genes involved in biofilm formation and DNA repair, we obtained detailed insights into the mechanism of bacterial cell death evoked by Cur-PDT.

## 2. Materials and Methods

### 2.1. Bacterial Strain and Culture Conditions

*B. subtilis* strain 168 from the Chinese Center of Industrial Culture Collection (CICC) was used in this study. This strain was stored at −80 °C with glycerol and incubated overnight at 37 °C. Enriched cultures were pooled into a 1.5 mL centrifugal tube and centrifuged at 4000× *g* and 4 °C for 7 min. The resulting cell pellet was washed with phosphate-buffered saline (PBS; Solarbio Biotech Co., Ltd., Beijing, China) to produce a cocktail of 10^7^–10^8^ CFU/mL. Prior to a bacterial colony count assay, the bacterial cell suspension was further diluted at different ratios and 100 µL bacterial suspensions were grown on LB-agar (Miller) plates for 16 h at 37 °C to determine corresponding cell numbers.

### 2.2. Preparation of Photosensitizer

Curcumin (>0.98%) was purchased from Solarbio Science & Technology Company. Moderate photosensitizers were dissolved in water and the pH of the resulting solution was adjusted to 7.40 with 1 M KOH. Samples were filtered (0.22 μm sterile filters) and incubated for 48 h in the dark at 4 °C.

### 2.3. Light-Emitting Diode (LED) System

The light source instrument with blue LEDs (60 V/5 A, 470 nm; Borui Instrument Equipment Co., Ltd., Suzhou, China) was enclosed in a black bag to prevent the entry of external light. The distance between the LED source and the bacterial solutions was adjusted to 5.0 cm. 

### 2.4. PDT Inactivation

Aliquots of the original bacterial suspension (10^7^–10^8^ CFU/mL) and the photosensitizer curcumin were added to a sterile centrifuge tube at a volume ratio of 1:1. In the experiment for detecting the effect of incubation time, 40 μmol/L curcumin and bacterial solution were placed together for 0, 15, 30, 45, 60, and 75 min at room temperature in the dark, after which the mixed solution was irradiated for 6 min by the LED light source instrument with the power of 120 W.

When analyzing the influence of light intensity, the mixed bacterial solution was treated with an LED light source at 0, 30, 60, 90, 120, and 150 W for 6 min. According to the results of the PDT sterilization effect, the irradiation was performed at a power of 120 W in subsequent experiments. Then, with curcumin concentrations of 10, 20, 30, 40, and 50 μmol/L, the bacterial solution was treated for 0, 3, 6, 9, 12, and 15 min, and the photodynamic sterilization effect was evaluated through a plate count. The plates were incubated at 37 °C for 12 h and the abundance of cells was expressed as log CFU/mL. Samples treated without light and photosensitizer, and with light or photosensitizer alone were chosen as a negative control, illumination control (L+/C−), and curcumin control (C+/L−), respectively.

### 2.5. ROS Measurement

The O11 probe (Baiaolaibo, Beijing, China) is cell-permeable and nonfluorescent but can be oxidized by ROS to produce green, fluorescent substances, which exist stably in bacterial cells. The intensity of green fluorescence can then be used to judge the change of ROS content in such cells. In this study, after curcumin-based photodynamic treatment, bacterial cells were immediately harvested and incubated with an O11 probe for 40 min in the dark at 37 °C. They were centrifuged at 5000 rpm for 10 min, the supernatant was discarded, washing was performed with phosphate-buffered saline (PBS) 2–3 times, and the bacteria were resuspended in PBS. The sample was observed under a fluorescence microscope (Zeiss, Germany) and ROS were analyzed using a fluorescence spectrophotometer with an excitation wavelength of 488 nm and an emission wavelength of 526 nm.

### 2.6. Oxidative Enzyme Detection

A total of 500 μL of phosphate buffer solution was added to the bacterial precipitation, which was then crushed in an ultrasonic cell crusher under conditions of an ice water bath at 300 W with ultrasound applied once every 3–5 s a total of four times. Then, the suspension was centrifuged at 10,000 rpm and 4 °C for 10 min, after which the supernatant, representing the enzyme extract, was transferred to a new tube and kept on ice. Following the manufacturer’s protocols for the superoxide dismutase (SOD) assay kit, catalase (CAT) kit, and glutathione peroxidase (GPX) kit (Jiancheng, Nanjing, China), the activities of SOD, CAT, and GPX, respectively, were determined and their relative activities were calculated according to the following formula: relative enzyme activity = enzyme activity of treated group/enzyme activity of untreated group. 

### 2.7. Propidium Iodide (PI) Staining

PI staing can indicate the permeability change of cell membrane. When the cell membrane is damaged, PI can cross intact plasma membrane and bind to the DNA in the cell. The bacteria were incubated with PI (Sigma, St. Louis, MO, USA) at a concentration of 10 μg/mL in the dark at 4 °C for 30 min. The bacterial precipitation was thoroughly washed in PBS by centrifugation at 5000 rpm for 10 min at 4 °C. The sample was observed under a fluorescence microscope (Zeiss, Oberkochen, Germany) and ROS were analyzed using a fluorescence spectrophotometer with an excitation wavelength of 488 nm and an emission wavelength of 630 nm.

### 2.8. Scanning Electron Microscopy (SEM) 

Bacterial suspensions were centrifuged for 5 min at 10,000× *g*. The resultant pellets were mixed with 2.5% glutaraldehyde and 4% formaldehyde buffer overnight at 4 °C. Subsequently, the cells were washed three times with PBS and fixed in 1% osmium tetroxide for 1–2 h. The fixed bacteria were dehydrated with 30–100% ethanol solution and treated with each concentration of 10, 30, and 50 μmol/L for 15 min. The sample was then treated with a mixture of ethanol and isoamyl acetate (*v*/*v* = 1/1) for 30 min, followed by pure isoamyl acetate for 1 h. After drying and coating, the specimens were observed by scanning electron microscopy (Hitachi US8200).

### 2.9. Transmission Electron Microscope (TEM) 

*B. subtilis* cells were fixed in buffered 2.5% glutaraldehyde and 4% paraformaldehyde. They were then washed three times with physiological saline to remove excess fixative, fixed in unbuffered 1% osmium tetroxide, and washed with physiological saline. After dehydration, the bacteria were treated with pure acetone for 20 min, mixed solution of embedding agent and acetone (*v*/*v* = 1/1) for 1 h, pure acetone for 20 min, a mixed solution of embedding agent and acetone (*v*/*v* = 3/1) for 3 h, and pure embedding agent overnight. After heating at 70 °C overnight, the samples were obtained and sliced into 70–90 nm sections by a LEICA EM UC7 ultra-thin microtome. The sections were stained with lead citrate solution, uranyl acetate, and 50% ethanol saturated solution for 5–10 min, respectively, and then viewed under a transmission electron microscope. 

### 2.10. RNA Extraction, Reverse Transcription, and RT-qPCR

According to the pre-experimental results (not shown), the bacterial cells were irradiated three times for 4 min each at intervals of 1 min, and obtained through multiple centrifugations. Then, total RNA was extracted using a Bacteria RNA Extraction Kit (Tiangen, Beijing, China) and resuspended in RNase-free water. The purity of RNA was determined by measuring the values of OD_230_, OD_260_, and OD_280_ using a NanoDrop 8000 spectrophotometer. The integrity of RNA was evaluated by 1.5% (wt/vol) agarose gel electrophoresis. cDNA was reverse-transcribed using the PrimeScript RT Reagent Kit (Takara). RT-qPCR was performed with the primers for the *TasA*, *UvrA*, and *RecA* genes on a Real-Time PCR system (Applied Biosystems, Foster City, USA). All RT-qPCR reactions were performed in a total volume of 20 μL. Cycling parameters included initial denaturation at 95 °C for 5 min, followed by 40 cycles of 95 °C for 30 s, 60 °C for 30 s, and primer extension at 72 °C for 30 s. The changes in relative gene expression were calculated by the 2^−ΔΔCT^ method. 

### 2.11. Statistics

All experiments were conducted in triplicate. Standard error was estimated for every experimental point and marked in the figures as an error bar. The data were analyzed with Origin 8.5 software (Origin Lab Corporation, Northampton, MA, USA). Data are expressed as mean ± SEM (N = 3 in each group, *p* < 0.05). 

## 3. Results

### 3.1. Antibacterial Efficacy of Photosensitizer-Mediated PDT against B. subtilis

**Figure 1:**
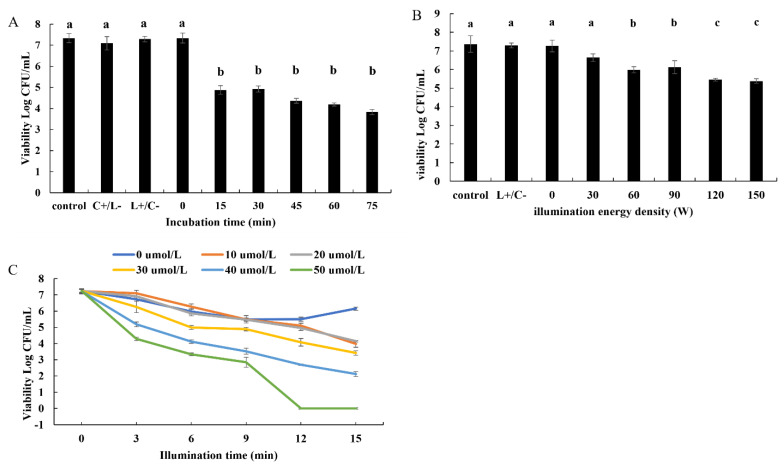
The viability of *B. subtilis* incubated with the different treatments. (**A**) *B. subtilis* was treated with 40 μmol/L curcumin−mediated PDT after the incubation with curcumin for 0, 15, 30, 45, 60, 75 min in the dark. (**B**) *B. subtilis* was treated 40 μmol/L curcumin−mediated PDT at 0, 30, 60, 90, 120, 150 W after the incubation for 15 min in the dark. (**C**) *B. subtilis* was treated with 0, 10, 20, 30, 40, 50 umol/L curcumin for 0, 3, 6, 9, 12, 15 min respectively at 120 W after the incubation for 15 min in the dark. Different lowercase letters indicate the significant differences (*p* < 0.05).

To determine the influence of curcumin-mediated PDT on the inactivation of *B. subtilis*, the effects of incubation time, light intensity, duration of irradiation, and photosensitizer concentration were analyzed. First, after co-culture with curcumin in the dark for different times, the number of surviving bacteria was determined. The results of plate counting revealed that the cell viability decreased rapidly in 15 min (4.88 log CFU/mL) and slowed down in the next 60 min (4.92, 4.36, 4.18, and 3.84 log CFU/mL at 30, 45, 60, and 75 min) (Figure 1A), and the difference from 15 min to 75 min was not significant. Therefore, 15 min of irradiation was chosen for use in the subsequent experiments. 

We further analyzed the inactivating effect of different light intensities on the bacteria, and found that the cell viability decreased significantly with increasing energy. As shown in Figure 1B, upon comparisons with the control and L+/C− groups, the cell viability at 30 W showed no obvious change. However, at 60 W, the surviving bacteria decreased by about 1.3 log CFU/mL, a level that did not differ from that at 90 W. When the power increased to 120 W and 150 W, the number of surviving bacteria decreased significantly (by 1.8 log CFU/mL), and there was little difference between them. Therefore, 120 W was selected as the light intensity for use in the subsequent experiments.

As shown in Figure 1C, the effects of different concentrations of photosensitizer and different durations of irradiation on the survival rate of *B. subtilis* were also found to be dose-dependent. When the irradiation time was used as a variable, the viability of bacterial cells subjected to Cur-PDT for the same time decreased with increasing curcumin concentration. When the irradiation time was 15 min, 10, 20, 30, 40, and 50 μmol/L curcumin could reduce the number of surviving bacteria by 3.25, 3.08, 3.82, 5.06, and 7.23 log CFU/mL, i.e., the survival rate decreased to 55.09%, 57.34%, 47.24%, 29.99%, and 0%, respectively. Moreover, at the same concentration, the survival rate decreased with increasing duration of irradiation. Taking the treatment with 50 μmol/L curcumin as an example, the cell viability was 7.24, 4.29, 3.34, 2.85, 0, and 0 log CFU/mL in 0, 3, 6, 9, 12, and 15 min, respectively. These findings suggested that 50 μmol/L curcumin could kill all cells upon exposure to the LED light source for 12 or 15 min. 

All of these results showed that curcumin-mediated PDT can effectively inactivate *B. subtilis*, in a manner dependent on the light intensity, duration of irradiation, and photosensitizer concentration. Moreover, it was shown that, after co-culture with 50 μmol/L curcumin for 15 min in the dark, the bacterial solution of *B. subtilis* could be killed totally with a 120 W LED blue light source for 12 min.

### 3.2. Effect of Cur-PDT on Cell Membrane Permeability

**Figure 2:**
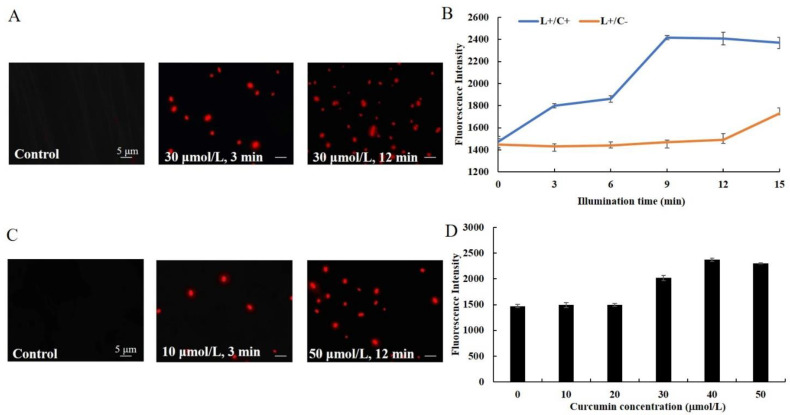
Changes of bacterial cell membrane permeability with different PDT treatments. The microscope images (**A**) and fluorescence intensity (**B**) of *B. subtilis* after Cur-PDT treatment with the illumination time of 3 and 12 min and 30 μmol/L curcumin by PI staining. The microscope images (**C**) fluorescence intensity (**D**) of *B. subtilis* after Cur-PDT treatment with curcumin concentrations of 10 and 50 μmol/L for 12 min by PI staining. The scale bar in the panel is five microns and all panels have the same magnification.

To distinguish the cells whose membranes were compromised from those that had intact membranes, a fluorescent staining assay was performed. Almost none of the cells before treatment (control) showed fluorescence. However, after 3 min of irradiation, some red fluorescent-stained cells were found. Meanwhile, when the treatment time was 12 min, almost all of the cells displayed the red fluorescence of PI (Figure 2A). Meanwhile, the fluorescence intensity of cells under different treatment conditions was determined. The results showed that, compared with the single light group (L+/C−), the fluorescence intensity of cells treated with Cur-PDT (L+/C+) increased with the duration of irradiation. At an irradiation time of 9 min, the fluorescence intensity of cells was about 1.6 times higher than that at 0 min, while between 9 and 15 min after irradiation it remained stable (Figure 2B). In addition, the cells subjected to 10 μM curcumin-mediated PDT for 12 min started to present red fluorescence, the level of which was much lower than that of 50 μM curcumin-mediated PDT-treated cells (Figure 2C). Besides, the fluorescence intensity of cells subjected to 30 μM curcumin-mediated PDT increased rapidly, and it was 1.6 times higher than that of untreated cells when the concentration of curcumin was 40 or 50 μmol/L (Figure 2D). These results showed that the permeability of the cell membrane increased significantly with increases in the duration of irradiation and photosensitizer concentration. 

### 3.3. Effect of Cur-PDT on Cell Morphology

**Figure 3:**
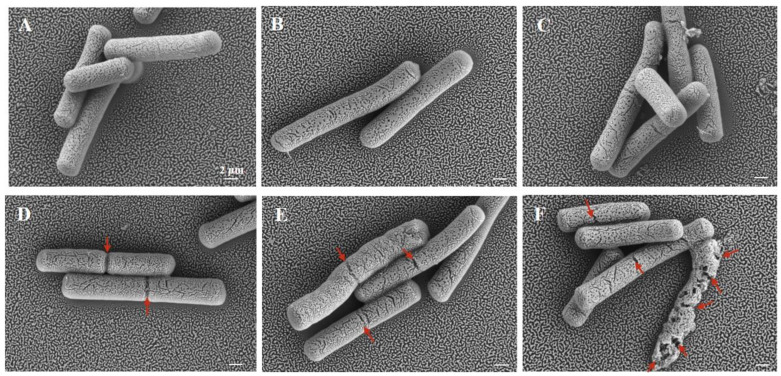
Scanning electron microscope image of *B. subtilis* after PDT treatment. (**A**) Untreated group; (**B**) single LED light group; (**C**) single 50 μmol/L curcumin group; (**D**) The treatment with 10 μmol/L curcumin for the illumination time of 12 min; (**E**) The treatment with 30 μmol/L curcumin for the illumination time of 12 min; (**F**) The treatment with 50 μmol/L curcumin for the illumination time of 12 min. The scale bar in the panel is 2 microns and all panels have the same magnification.

To further observe the cell structural changes of bacteria, cell morphology was visualized by SEM and TEM. The SEM images revealed different degrees of damage on the surface of the bacterial cells with concentrations of curcumin ranging from 10 to 50 μmol/L (Figure 3D–F), in contrast to the untreated cells and cells treated only with irradiation and only with curcumin (Figure 3A–C). When the concentration of curcumin was 10 μmol/L, the cells displayed coarse grooves reflective of damage and cracks on their surface (Figure 3D). The damage became more severe upon treatment with increasing concentrations of curcumin. Specifically, at a concentration of 30 μmol/L, the cracks became wider and deeper (Figure 3E), and the cells were even deformed or crushed at 50 μmol/L (Figure 3F). 

**Figure 4:**
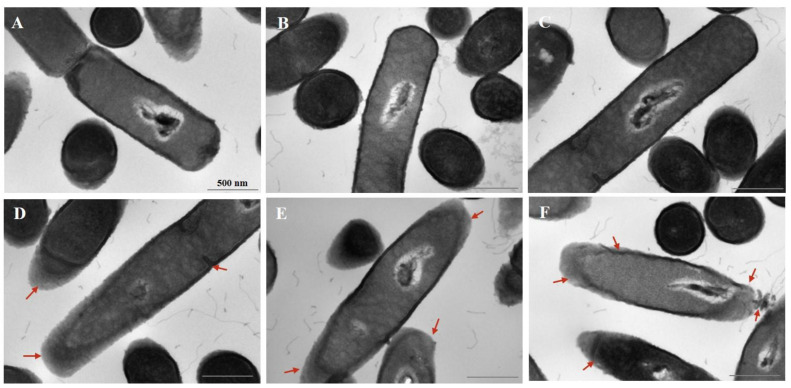
Transmission electron microscope image of *B. subtilis* after PDT treatment. (**A**) Untreated group; (**B**) single LED light group; (**C**) single 50 μmol/L curcumin group; (**D**) The treatment with 10 μmol/L curcumin for the illumination time of 12 min; (**E**) The treatment with 30 μmol/L curcumin for the illumination time of 12 min; (**F**) The treatment with 50 μmol/L curcumin for the illumination time of 12 min. The scale bar in the panel is 500 nm and all panels have the same magnification. Red arrow indicates the damaged cell membrane.

In addition, the TEM images showed that untreated *B. subtilis* cells were rod-shaped with clear edges, an intact cell wall, dense and uniform cytoplasm and nuclear morphology, and no separation of the plasma wall (Figure 4A). The cell morphology of the light alone control group (L+/C−) (Figure 4B) and curcumin alone control group (C+/L−) (Figure 4C) did not change significantly, compared with that of the untreated cells. However, upon treatment with 10 μmol/L curcumin-PDT for 12 min, the edge of the cell wall was clearly blurred (Figure 4D). With an increase in the concentration of curcumin to 30 μmol/L, the destruction of the cell wall was aggravated, the cytoplasm and the cell wall began to separate, and the nuclear morphology changed (Figure 4E). Finally, at a concentration of 50 μmol/L, the cell wall structure at both ends of the cells was completely destroyed, severe plasmolysis developed, the morphology of the central nucleus changed significantly, and the cell contents flowed out in large quantities (Figure 4F). 

All of these findings suggested that, with increasing curcumin concentration, the morphology of cells underwent significant changes, and cell membrane permeability and structure were seriously damaged.

### 3.4. Changes of Redox State in Cells after Curcumin-PDT Treatment

**Figure 5:**
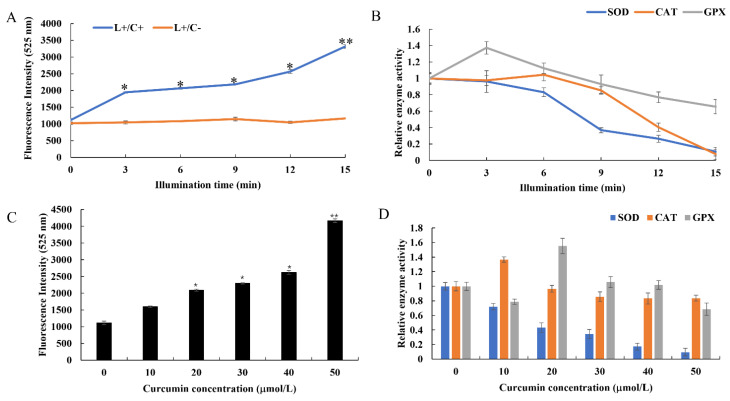
Effect of Cur-PDT treatment on redox state of *B. subtilis* in different conditions. Changes of ROS production under Cur-PDT treatment with different illumination time (**A**) and curcumin concentrations (**C**); Changes of antioxidant enzyme activity under Cur-PDT treatment with different illumination time (**B**) and curcumin concentrations (**D**). Asterisk indicates the significant differences (*, *p* < 0.05; **, *p* < 0.01).

To determine whether curcumin would produce ROS upon irradiation, we used the O11 fluorescent probe to indicate ROS production. The suspensions of *B. subtilis* were incubated with 30 μmol/L curcumin under 120 W irradiation for 0, 3, 6, 9, 12, and 15 min. Upon measuring the fluorescence, we found that the amount of ROS increased with increasing irradiation time. Curcumin-mediated PDT led to the production of approximately two-fold and three-fold more ROS upon irradiation of *B. subtilis* for 3 and 15 min, respectively, compared with the level in the unirradiated control (0 min) (Figure 5A). In addition, following the ROS production assay conducted after curcumin-PDT treatments with different concentrations of 0, 10, 20, 30, 40, and 50 μmol/L, the results indicated that there were no significant differences in ROS production in the low-dose (10 μmol/L) and irradiation-only groups (0 μmol/L) (Figure 5C). However, with increasing curcumin concentration, the level of ROS production gradually increased. The ROS production was 2–4 times higher upon treatment with 20–50 μmol/L curcumin-PDT than that in the irradiation-only group (0 μmol/L). These results for the Cur-PDT groups suggested that PDT treatment would induce ROS production in *B. subtilis*, and the amount of ROS generated was directly proportional to the irradiation time and curcumin concentration. 

Under physiological conditions, the oxidation and antioxidant systems in organisms maintain a dynamic balance. However, upon exposure to harmful stimulation, ROS increase in the organism, potentially causing oxidative stress. At this time, as an effective defense system against such stress, antioxidant enzymes, such as SOD, CAT, and GPX, undergo changes in their activity. Therefore, we measured the changes in SOD, CAT, and GPX activity in bacterial cells in order to judge the oxidative damage suffered by *B. subtilis* due to curcumin-PDT. Upon treatment for 3 min, the activity of SOD in bacterial cells increased significantly, while the activities of GPX and CAT increased slightly. However, when the irradiation time was extended to 6 min, the activities of SOD and GPX began to be inhibited, while the activity of CAT remained slightly increased. With further extension of the irradiation time, the activities of all three enzymes decreased significantly. Upon treatment for 15 min, the relative activities of CAT and SOD were close to zero (Figure 5B). Meanwhile, with increasing curcumin concentration, the activities of the three enzymes showed different trends. At a concentration of 10 μmol/L, the activities of both SOD and GPX decreased, while CAT activity increased. However, at 20 μmol/L Cur-PDT, CAT began to decline slightly, while GPX was first elevated and then gradually decreased. Interestingly, SOD continuously exhibited a gradual decline (Figure 5D). 

The above results show that, with increasing irradiation time, the level of ROS in *B. subtilis* increased markedly, while the activities of ROS-scavenging enzymes SOD, CAT, and GPX gradually decreased. Excessive ROS strongly inhibited the activities of three enzymes, suggesting that *B. subtilis* may be seriously damaged by oxidation. Moreover, its oxidative stress response was shown to occur in a manner dependent on irradiation time and photosensitizer concentration.

### 3.5. Regulatory Effects of Cur-PDT on Membrane Structure and DNA Repair System at the Transcriptional Level

In a preliminary experiment, we found that the total RNA of *B. subtilis* was degraded significantly even with 10 μmol/L curcumin-mediated PDT applied for 9 min (data not shown). We speculated that curcumin-mediated PDT could have a strong and rapid killing effect on *B. subtilis* cells, achieved via damage to bacterial mRNA. Therefore, we reduced the concentration of curcumin to 2.5 μmol/L and irradiated *B. subtilis* cells for 12 min discontinuously, so as to slow down the generation of bacterial oxidative stress through reducing the curcumin concentration and shortening the illumination time. Then, the bacterial cells were obtained and subjected to qPCR.

**Figure 6:**
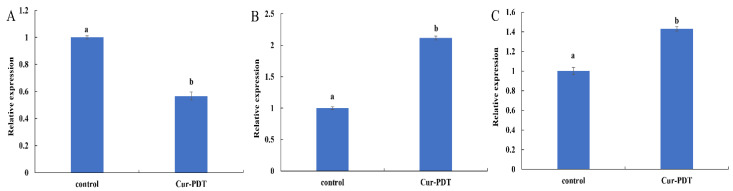
The expression levels of TasA (**A**), RecA (**B**) and UvrA (**C**) mRNAs of *B. subtilis* with 2.5 μmol/L curcumin-mediated PDT treatment. Different lowercase letters indicate the significant differences (*p* < 0.05).

Extracellular protein TasA is a component of *Bacillus* biofilm that is closely related to the stability of the biofilm structure. As shown in Figure 6A, the expression of TasA mRNA in *B. subtilis* cells subjected to curcumin-PDT was significantly downregulated to 0.57-fold that of the untreated control group (*p* < 0.05), indicating that the synthesis of this extracellular protein was affected and thus the bacterial biofilm structure was damaged. In addition, ROS generated by PDT treatment may damage bacterial DNA, so the DNA-binding (recognition) protein UvrA and the DNA damage repair protein RecA were used as indicators to judge the impact of PDT treatment on *B. subtilis* DNA. The results shown in Figure 6B,C indicate that, after PDT treatment, the expression of RecA and UvrA in *B. subtilis* was significantly upregulated to 2.12 and 1.43 times the levels of the control group (both *p* < 0.05), respectively. All of these findings indicated that, during PDT treatment, the DNA of the bacteria was damaged and the response system of DNA repair was induced.

## 4. Discussion

Photodynamic sterilization technology is still in its infancy in research for the food industry. Many issues still require intensive study, such as the selection of edible photosensitizers, the application of bacterial strains, and the conditions of PDT sterilization. In this study, we chose curcumin as a photosensitizer and used the photodynamic equipment of an LED light source to analyze the effects of four factors on the killing of *B. subtilis*: duration and intensity of irradiation, incubation time, and photosensitizer concentration. The results showed that 50 μmol/L curcumin-mediated photodynamic sterilization technology could effectively kill all *B. subtilis* within 15 min. This killing effect would be extremely useful for actual food production and is of great significance for the food processing industry. We also explored the mechanism by which curcumin-mediated photodynamic technology killed *B. subtilis*. The results showed that it led to the substantial production of ROS in bacterial cells, followed by damage to DNA, rupture of the cell membrane, and bacterial death.

In the present study, we found that the sterilizing effect of curcumin-mediated PDT on *B. subtilis* was dependent on the curcumin concentration, duration of irradiation, and light intensity. Chen et al. found that 1 μmol/L curcumin-mediated PDT treatment for 5 min reduced the abundance of *V. parahaemolyticus* by ~108 log CFU/mL. Moreover, 20 μmol/L Cur-PDT was found to eradicate *V. parahaemolyticus* biofilm under light irradiation for 60 min [21]. Moreover, 50 μmol/L Cur-PDT decreased the level of *V. parahaemolyticus* from 5.2 to 3.7 log CFU/g on cooked oysters when applied for 30 min, while 100 μmol/L Cur-PDT inactivated it to a level that was undetectable [23]. These results further indicate that PDT can inactivate microorganisms in a manner dependent on the duration of irradiation and the photosensitizer concentration. 

Our results showed that curcumin-mediated PDT can inactivate *B. subtilis* effectively by inducing the production of a large amount of ROS in bacterial cells. Previous studies have proven that, after the excitation of photosensitizer through the absorption of light, energy transfer with the surrounding biomolecules or oxygen molecules occurs to produce ROS, which are considered to be the factor directly causing bacterial damage [6]. Qi et al. verified that hypocrellin A-mediated PDT could significantly increase ROS to inhibit cell apoptosis, which was reversed by reducing the amount of ROS by treatment with a free radical quencher [24]. In this experiment, we found that, compared with the group without photosensitizer and only exposed to light (L+/C−), the ROS production increased significantly with increases of the duration and intensity of irradiation, and photosensitizer concentration. This could indicate that prolonged irradiation would induce continuous excitation of the photosensitizer, after which the surrounding increasingly abundant oxygen molecules would be continuously transformed into ROS. In addition, the increase of photosensitizer concentration would promote ROS production under the same light conditions upon an increase in the source of singlet oxygen. Accumulated ROS would invade the bacterial cell, which could finally lead to cell death [25].

Accumulated reports have shown that a large amount of ROS would lead to an imbalance of the redox system in cells, which has always been considered a main cause of PDT treatment [26]. In this study, the changes of the activity of three antioxidant enzymes (SOD, CAT, and GPX) in bacteria were also measured. The results showed that, with increasing irradiation time, the production of ROS gradually increased. At the same time, the activities of GPX and CAT enzymes increased significantly at 3 and 6 min, respectively, and then decreased, while SOD consistently showed a gradual decrease (Figure 5A,C). Moreover, the same findings were obtained when the concentration of curcumin was changed (Figure 5B,D). In the early stage of Cur-PDT treatment, the bacterial defense system was activated by ROS production, and the activities of CAT and GXP increased to varying degrees, which can remove excess ROS and maintain the dynamic redox balance in cells. However, with prolonged irradiation and increased curcumin concentration, the production of ROS was too high to be scavenged, which led to breakdown of the redox state in cells and significant reductions in the activity and function of antioxidant enzymes. In a previous study, dimethyl phthalate was used to induce oxidative stress in *E. coli* and *B. subtilis*, and their enzyme activities were reported to be promoted at a low concentration of it and inhibited at a high concentration, which is consistent with our results [27]. This can be explained by the fact that bacterial death often involves the sensitization of cells by the extracellular environment and is also commonly a result of cumulative damage [28].

Accumulating studies have found that ROS contribute to oxidative damage, mainly including cell membrane damage, intracellular biopolymer leakage, and cytoplasmic degeneration. Therefore, to confirm this damage, we observed the effect of ROS on the internal and external morphology of cells by TEM and SEM. A higher level of ROS production was shown to be associated with higher permeability of the cell membrane. In terms of the changes in external morphology observed by SEM, it was observed that the bacteria in the control group were still intact after the Cur-PDT treatment, but they displayed the phenomenon of surface perforation. Moreover, the group treated with high-concentration PDT released more ROS and almost all of the cells were completely ruptured and died. Furthermore, the internal morphology of the bacteria as revealed by TEM showed that PDT treatment resulted in various types of damage to the cells, including severe disruption of the cell membrane, separation of the plasma wall, massive loss of intracellular contents, and changes in nuclear morphology. The findings also suggested that the bacterial cell membrane is always the common target of oxidative damage [27]. This can be explained by the fact that expression of the cell membrane synthesis gene TasA was suppressed upon PDT treatment. TasA, the major proteinaceous component of *B. subtilis* extracellular matrix, can form long protein fibers that contribute to the superstructure of the biofilm matrix and are needed for biofilm integrity [29,30]. Indeed, many studies in the literature have demonstrated that PDT can destroy the biofilm of microorganisms to display the sterilization effect [31]. Moreover, several studies have shown that PDT treatment can damage the cell membrane by destroying its structural integrity. This inhibits the viability and growth of bacterial biofilm, e.g., in *Pseudomonas aeruginosa*, *Propionibacterium acnes*, and *Staphylococcus aureus* [32,33,34]. The outer membrane structure maintains a stable intracellular environment by controlling the entry and exit of substances inside and outside the cell. However, upon PDT treatment, ROS first destroy the structure of the cell membrane, allowing more photosensitizers to enter the cell. Upon continuous stimulation, more intracellular ROS are produced and accumulate, causing oxidative damage to the nucleus and other organelles inside the cell, and eventually leading to leakage of the cell contents and cell death. 

In addition, given that DNA forms the basis of normal physiological activities and reproduction of bacteria, damage to it can also contribute to bacterial death. Indeed, ROS have been reported to cause irreversible damage by interacting with important biomolecules, such as lipids, proteins, and DNA, ultimately leading to cell death [35]. In a previous study, PDT combined with any of the four tetracyclines plus chitosan killed *Clostridioides difficile* efficiently by causing DNA damage [36]. In addition, 5-aminolevulinic acid-mediated photodynamic therapy has been reported to inactivate *Sporothrix globosa* due to the generation of reactive oxygen species and DNA damage [37]. Moreover, Sabarinathan et al. [38] found that DNA fragmentation is the main cause of MCF-7 cell apoptosis due to new porphyrin photosensitizer-mediated PDT. In our study, the expression of the DNA repair genes RecA and UvrA in *B. subtilis* was upregulated after Cur-PDT treatment, which indicated that the bacterial DNA repair response system had been stimulated. This further proved that PDT would promote DNA damage in cells, resulting in bacterial death. Therefore, we speculate that the death of *B. subtilis* caused by Cur-PDT in this study may also be related to DNA damage.

## 5. Conclusions

Photodynamic treatment using curcumin as a photosensitizer appears to be a promising approach for inactivating *B. subtilis*. Our results demonstrated that 50 μmol/L Cur–PDT decreased *B. subtilis* (10^7^–10^8^ CFU/mL) to non-detectable levels upon irradiation at 120 W for 15 min. Additionally, we showed that the sterilizing effect of curcumin is achieved by inducing the production of ROS and the subsequent induction of oxidative stress in bacteria. Further studies on the application of Cur-PDT treatment in food systems and for killing particular bacterial strains are still needed.

## Data Availability

Not applicable.
